# Sequential effects in Olympic synchronized diving scores

**DOI:** 10.1098/rsos.160812

**Published:** 2017-01-25

**Authors:** Robin S. S. Kramer

**Affiliations:** Department of Psychology, University of York, York YO10 5DD, UK

**Keywords:** sequential effects, judgements in sports, diving, assimilation, contrast

## Abstract

When judging performances in a sequence, the current score is often influenced by the preceding score. Where athletes are perceived to be similar, a judgement is assimilated towards the previous one. However, if judges focus on the differences between the two athletes, this will result in a contrasting influence on their scores. Here, I investigate sequential effects during synchronized diving events at the 2012 and 2016 Olympic Games. Although previous research found assimilation in scores of gymnasts, the current data showed contrast effects—current scores benefited from following a poor performance but were at a disadvantage if they followed a high-scoring performance. One explanation may be that the processes involved in judging synchronized pairs results in a focus on the differences between athletes, producing a contrast effect across dives. That the specific direction of this sequential bias may depend on the particular sport has implications for how judges might approach their roles in a context-dependent manner, as well as how such biases should be addressed.

## 1. Introduction

Many judgements in life are supported by the use of technical instruments, where an objective measurement can be achieved—a ruler gives us the length of a line, or scales will determine our weight. In these situations, we can be fairly sure that such measures are (relatively) accurate and the role of subjective judgements has been minimized. However, we are often asked to quantify something beyond the scope of instrumentation and we are required to use our own opinions, prior experience and judgements. For example, we might be asked how much we enjoyed our meal or how attractive a particular celebrity is. In these situations, there is no (objectively) correct answer, simply our own personal opinion. Even when adhering to specific criteria or guidelines (e.g. when judging artistic gymnastics at the European Championships), judges' scores can still differ significantly from each other [1]. In these circumstances, despite the use of scoring guidelines and often extensive training prior to qualification as a judge, people's opinions will always vary.

Human behaviours are biased in predictable ways. For example, researchers find an advantage for the first and last items in a sequence during later recall (known as the *primacy* and *recency* effects, respectively; [2]). Indeed, these memory effects are mirrored in decision-making, where contestants who perform first or last in a competition are more positively evaluated [3], as are wines tasted in a sequence [4]. When several decisions are made consecutively, these serial position effects tend to result in an advantage for those who perform later in the competition [5–8]. In addition to these ‘location in the overall sequence’ biases, researchers have also demonstrated particular sequential effects that operate locally. Simply, the directly preceding trial (or trials) can influence one's judgement of the current trial, no matter where in the sequence it takes place. This type of bias has been found in a variety of contexts, including the performance judgements of Olympic athletes [9], estimates of item prices [10], ratings of students' essays [11] and perceptions of attractiveness in both the laboratory [12] and while speed dating [13].

The direction of the previous judgement's influence appears to depend on the similarity between the previous and current trials. When the two neighbouring trials (athletes, stimuli, etc.) are perceived to be similar, the selective accessibility model [14] suggests that judges will focus on further similarities (hypothesis-consistent evidence), resulting in an assimilation effect. If the previous athlete receives a high score, for instance, then this will increase the evaluation of the current athlete under consideration. However, if the two trials are sufficiently dissimilar, the opposite will happen. Judges will focus on further dissimilarities, producing a contrast effect—the high-scoring performance of the previous athlete will result in a lower evaluation of the current athlete.

Some researchers have also argued that timing is important when predicting the direction of after-effects [15]. Typically, assimilation is associated with very brief presentations (perhaps in the order of fractions of a second), while longer durations result in contrast effects [16]. Indeed, assimilation along various perceptual dimensions has been shown for 300 ms stimulus presentations [17,18]. However, assimilation towards a previously given rating appears unaffected by increases in presentation duration [19], and assimilation effects have been found when presentations were unlimited and responses were self-paced [12,20]. Interestingly, longer intervals between adjacent ratings may decrease the magnitude of this assimilation [11].

### 1.1. Sequential judgements in sports

Judges in sporting competitions are often required to assess the performances of numerous athletes consecutively (e.g. in diving, gymnastics, synchronized swimming, ski jumping, figure skating, etc.). Crucially, these sequential performance judgements can be influenced by a range of factors above and beyond the particular performance under consideration. Although most sporting competitions make allowances for the fact that judges' scores will naturally vary (e.g. by averaging across multiple judges, dropping the highest and lowest scores, etc.), they often lack additional procedures for addressing various human biases. These may include a nationalistic bias, where judges give higher scores to competitors from their own countries [21,22], a difficulty bias, where athletes attempting more difficult routines receive higher execution scores [23], or a bias to conform after receiving feedback about other judges' scores [24,25].

Here, I focus on the sequential effects biases described above, where the previous trial can influence the current one. This type of bias may be particularly prevalent in the judging of sports in which decisions are often made under suboptimal conditions—requiring high levels of concentration over hours of judging, applying complex criteria when making decisions, and with performances sometimes lasting only a second or two. Importantly, while television viewers are often shown slow-motion replays of Olympic performances (e.g. dives), judges do not have access to these and must generate scores based on their viewing in real time. Under such conditions, judges may rely on simple comparison heuristics [26].

Research suggests that the degree of perceived similarity between the previous athlete and the current one is important in determining whether an assimilation or contrast effect will take place. Damisch and colleagues [9] found that scores were assimilated during gymnastics events at the 2004 Olympic Games—for a pair of consecutively starting gymnasts, a good performance by the first athlete will improve the score given to the second athlete. In follow-up studies carried out under experimental conditions, the authors also manipulated the perceived similarity between athletes using either a procedural priming task or by altering the apparent nationalities of the athletes to be either the same as each other or different. As predicted, both assimilation (due to high similarity) and contrast (due to low similarity) effects were produced, depending on the particular condition in which participants appeared. Importantly, in the real-world context of the Olympic Games, similarities between athletes were apparently more salient in that only assimilation effects were detected.

However, more recently, a study by Rotthoff [7] examined data from the 2009 World Artistic Gymnastics Championships (held in London, England) and found no evidence of sequential order effects, although an overall order bias (where later performers scored higher) was apparent. It remains unclear as to why assimilation effects appeared in 2004 but were not present in a similarly elite-level competition that took place five years later. This represents one of the motivations for this work.

### 1.2. This research

In this research, I investigated whether sequential judgements in Olympic synchronized diving events led to either assimilation or contrast effects (or neither). If such biases were found, this would represent a useful replication and extension of previous work examining Olympic gymnastics results [9]. However, there is also reason to predict the absence of sequential effects [7] and so further evidence, exploring a different set of sporting events, provides useful additional data that may bring some clarity to the field. Given that assimilation effects were identified in the study of gymnastics judges at the 2004 Olympics, I make the same prediction here for diving events.

Why predict assimilation, particularly in a synchronized diving competition? Assimilation relies on judges perceiving competitors as similar. In general, people appear to focus initially on ways in which two items are similar rather than different [27], leading them typically towards similarity testing [14]. This may be because focusing on similarities is a faster and more efficient style of thinking [26,28]. Of course, in the particular case of synchronized divers, there are numerous similarities that may prime this type of focus. For example, athletes during an event are the same sex, approximately similar in age, wearing similar clothing, and carry out many of the same diving routines. Indeed, the very nature of synchronized diving means that each pair of divers (competing together) strives to be identical in every way. Perhaps, this in itself primes judges to focus on similarities rather than differences.

The advantage of analysing data collected from real-world sporting competitions is the high degree of ecological validity that this confers. These scores were actually awarded to competitors and resulted in the winning (or losing) of Olympic medals. There is therefore no question about the applicability of the findings to contexts outside the laboratory. Experimental studies, by contrast, may focus on teasing apart particular explanations through the presentation of a single pair of performances rather than a full-length competition sequence, for example (e.g. Studies 2 and 3 of [9]). Whether decision-making is comparable in the two situations remains untested.

Of course, these types of field studies also have their disadvantages. Here, the results will be correlational and cannot inform as to the causal relationship between previous and current performance judgements in a sequence. I cannot, for instance, determine whether it is the degree of perceived similarity between pairs of athletes that influenced subsequent judgements. However, the real-world nature of the data provides us with a rare chance to investigate how judges actually behave on the world's stage.

## 2. Material and methods

### 2.1. Dataset

I collected judges' scores during the finals of the synchronized diving events held at both the 2012 (London, England) and the 2016 Olympic Games (Rio de Janeiro, Brazil). These included synchronized 10 m platform and synchronized 3 m springboard events, for both men and women. Women's synchronized pairs perform five dives during their competitions, while men's pairs perform six, and 11 judges score each dive in all cases. In total, this resulted in 1936 judgements across all events for each Games. Taken together, this provided 3872 scores for analysis (see the electronic supplementary material).

Synchronized diving events were chosen because the diving order is determined by random computer selection rather than previous performance/rankings. That is, the order of the pairs is random in the first round, and then remains unchanged for subsequent rounds. The eight pairs that take part in the Olympic final (there are no prior heats) comprise the top three from the World Championships, the top four from the World Cup and the host nation (here, Great Britain in 2012 and Brazil in 2016).

Eleven judges score each dive—three score the execution of diver A, three score the execution of diver B and five score the synchronization of the pair. Each judge provides a score between 0 and 10 points (in 0.5 point increments), and these are combined using a specific formula to produce the overall score for the dive. The panel of judges score the dive immediately after it has taken place without communicating with each other. The execution judges only consider the technique and execution of the particular diver they have been assigned to, while the synchronization judges only consider the overall impression of the synchronization of the two dives (the approach and take-off, the coordinated timing of the entries, etc.). Importantly, the judges must not be influenced by any other factors [29]. This means that the degree of difficulty of a dive should not be a consideration when scoring (because the difficulty itself is subsequently factored into the formula, which produces the final score). Therefore, although more difficult dives are performed in later rounds, the judges' raw scores should not necessarily reflect this. However, more difficult dives include more components and hence an increased likelihood for divers to make mistakes.

### 2.2. Statistics

In order to investigate whether the preceding divers influenced judges' current scores, I performed several Pearson product–moment correlations, pairing each judge's score with the score they gave for the previous dive. These *N*– 1 correlations, as they are known, measure how much judges use the dive they have just scored as a comparison standard when judging the current dive. A positive correlation represents an assimilation effect, where, for example, a high score given on the preceding dive increases the score given to the current dive. Alternatively, a negative correlation represents a contrast effect, where, for example, a low score on the preceding dive increases the current dive's score.

While previous researchers have investigated the potential influence of scores given two or even three judgements prior to the current one [9], I focus here on only the last dive just prior to the current dive. This is because each round in diving consists of only eight dives, and so very few of these could be analysed to determine the influence of dives taking place so much earlier. As I show below, it was important with the current dataset to consider each round of dives separately in order to minimize the influence of any serial position effects over the whole event.

When combining multiple correlations (across rounds and judges), I use meta-analyses to calculate mean weighted effect sizes and 95% confidence intervals. Although I found evidence of heterogeneity across correlations in only some instances (considering Cochran's *Q* statistic), I report random effects models in all cases. Here, correlations are combined across different judges, who are also performing different types of decisions (single diver execution versus pair synchronization), providing a theoretical rationale for this approach. Further, random effects are considered more realistic and many recommend their use in all situations [30]. Finally, fixed effects models are simply a special case of random effects models, where the population variance happens to be zero [31].

In line with modern approaches to statistical reporting where effect sizes are preferred over traditional null-hypothesis significance testing [32], I only provide effect sizes and confidence intervals here. For readers who are still interested in the question of statistical significance, 95% CIs that do not include zero are significantly different from zero at an alpha level of 0.05.

## 3. Results

For each event, I carried out a correlational analysis including all scores, combining all judges and rounds (correlating 429 pairs of values for each women's event and 517 for each men's event). The results can be seen in the first column of values of table 1.

**Table 1.** A summary of the correlational analyses across all dives. Square brackets represent 95% confidence intervals. *cf* means controlling for the variable in the partial correlation.

			serial position and scores		difficulty and scores	
Olympic games	event	all scores, *r*	*r*	*cf* difficulty, partial *r*	*r*	*cf* serial position, partial *r*
London 2012	women's 10 m	0.28 [0.19, 0.36]	−0.47 [−0.51, −0.43]	−0.05 [−0.15, 0.05]	−0.52 [−0.55, −0.48]	−0.26 [−0.34, −0.17]
London 2012	women's 3 m	0.15 [0.06, 0.24]	−0.40 [−0.44, −0.37]	−0.08 [−0.13, −0.02]	−0.43 [−0.47, −0.40]	−0.19 [−0.25, −0.13]
London 2012	men's 10 m	0.09 [0.00, 0.17]	−0.34 [−0.40, −0.27]	0.06 [0.00, 0.12]	−0.44 [−0.50, −0.37]	−0.30 [−0.35, −0.24]
London 2012	men's 3 m	0.22 [0.14, 0.30]	−0.42 [−0.45, −0.39]	−0.09 [−0.12, −0.05]	−0.46 [−0.49, −0.42]	−0.21 [−0.25, −0.17]
Rio 2016	women's 10 m	0.27 [0.18, 0.36]	−0.34 [−0.40, −0.27]	0.05 [−0.04, 0.14]	−0.41 [−0.45, −0.37]	−0.26 [−0.32, −0.19]
Rio 2016	women's 3 m	0.29 [0.20, 0.37]	−0.40 [−0.44, −0.36]	−0.15 [−0.18, −0.12]	−0.39 [−0.42, −0.35]	−0.09 [−0.11, −0.07]
Rio 2016	men's 10 m	0.09 [0.00, 0.17]	−0.27 [−0.31, −0.23]	0.06 [−0.01, 0.13]	−0.35 [−0.40, −0.29]	−0.24 [−0.32, −0.16]
Rio 2016	men's 3 m	0.04 [−0.04, 0.13]	−0.46 [−0.49, −0.43]	−0.18 [−0.23, −0.13]	−0.44 [−0.49, −0.40]	−0.11 [−0.17, −0.04]

The analyses revealed positive correlations between the target divers' scores (remember that each performance is carried out by two divers) and the scores of the divers right before them, in line with previous research [9]. This assimilation effect means that the score of a diving pair increases with increasing scores of the pair that dived directly before them, but it also means that their score decreases with decreasing scores of the preceding pair.

### 3.1. Serial position effects

Problematically, there is evidence to suggest that, as competitions progress, scores can be affected by the order of appearance. These serial position effects mean that competitors tend to receive an advantage simply from appearing later in the competition [5,6,8]. To test this idea, for each of the 11 judges, the scores they gave throughout the competition were correlated with the order they were given in (serial position: 1–40 for women's events and 1–48 for men's). I then performed a meta-analysis on these 11 correlations in order to calculate the mean weighted effect size and its 95% confidence intervals across judges. As table 1's second column of values illustrates, I found negative correlations for serial position effects, suggesting that scores decreased as the competition progressed. Although this result is in the opposite direction to previous research [7], it may be explained by the increase in difficulty in synchronized dives later in the competition. While judges are instructed to give scores that are independent of the degree of difficulty for a given dive (see §2.1), it seems likely that the divers make more execution and synchronization errors with more difficult dives. As such, lower scores would be given in later rounds. (The first two rounds of dives always have the lowest degree of difficulty, with divers free to choose which dives to perform in subsequent rounds.)

If divers perform harder dives and consequently received lower scores in later rounds, this would also produce a pattern in the opposite direction to that predicted by the difficulty bias (higher execution scores given to more difficult routines) found in gymnastics [23]. To test this idea, for each of the 11 judges, the scores they gave throughout the competition were correlated with the difficulties of the dives (which are predefined in the governing body's rules and regulations; [29]). I then performed a meta-analysis on these 11 correlations as above. As table 1's fourth column of values illustrates, I found negative correlations with difficulty in all cases—the higher the degree of difficulty, the lower the score given.

To complicate matters, difficulty and serial position are confounded because the least difficult dives always take place in the first two rounds. Therefore, as serial position increases (later dives), so does the difficulty (harder dives). In order to investigate which factor better explains the decrease in scores later in the competition, I carried out partial correlational analyses. First, I correlated serial position and scores, controlling for the dives' difficulties (tables 1's third column of values). Next, I correlated difficulties and scores, controlling for the serial position (tables 1's fifth column of values). While difficulty continues to show a negative (although reduced) relationship with scores after controlling for serial position, I found that the relationship between serial position and scores is virtually absent after controlling for difficulty. This suggests that difficulty strongly mediates (although perhaps not completely) the relationship between serial position and scores [33]. Therefore, increasing difficulty in later rounds better explains why scores decrease as the competition progresses.

### 3.2. Sequential effects

If higher scores are given in earlier rounds and lower scores are given later in the competition, this would explain the positive correlations found initially when all rounds were combined. Each score is paired with the one from the previous dive, and so earlier (higher) scores are paired with each other, as are later (lower) scores. To take this into account, I next analyse each round of eight dives separately. Interestingly, the first dive of each round (after the first round) is preceded by a short between-rounds interval. Although only a few minutes in length, this interval between judgements is longer than the typical time between dives within the round itself. I therefore consider these judgements separately because it is possible that a longer time interval might affect how the last dive influences the current one.

For each judge, the between-rounds and within-rounds scores were analysed separately. In the between-rounds analyses, four (women's events) or five (men's events) pairs of values were included in each judge's correlation (one less than the number of rounds). A meta-analysis was then carried out on these 11 correlations. In some cases, fewer correlations were analysed because judges showed no variation in their scores for these particular dives, and so no correlations could be calculated.

For the within-rounds analyses, seven pairs of values were included in each correlation (one less than the number of dives in a round). Each judge therefore provided five (women's events) or six correlations (men's events), resulting in meta-analyses that included either 55 or 66 correlations, respectively. Note that for each judge, rounds were treated separately, rather than incorporating all within-round scores in a single correlation, because lower scores are given in later rounds and this alone (as discussed above) would result in positive correlations.

Analysis of the between-rounds pairs of scores determines the influence of the last score given in a round on the first score given in the next round. Analysis of the within-rounds pairs of scores quantifies the influence of the preceding score given in a round on the current score given in that same round. The results of these meta-analyses are presented in table 2 (first and third columns of values). It is clear that within rounds, judges' scores typically show contrast effects, although the women's events in 2016 failed to show evidence of this effect. However, between rounds, evidence of sequential effects is more mixed. In general, effects are positive but confidence intervals are large and often include zero. Interestingly, in the women's 3 m springboard at both Olympics, there appears to be a strong assimilation effect, although it is not clear why this would be the case.

**Table 2.** A summary of the correlational analyses for sequential effects, separating dives within and between rounds. Square brackets represent 95% confidence intervals. *cf* means controlling for the variable in the partial correlation.

		sequential scores within rounds		sequential scores between rounds	
Olympic games	event	*r*	*cf* difficulty, partial *r*	*r*	*cf* difficulty, partial *r*
London 2012	women's 10 m	−0.33 [−0.42, −0.23]	−0.37 [−0.47, −0.26]	0.09 [−0.32, 0.47]	−0.95 [−1.00, 0.35]
London 2012	women's 3 m	−0.19 [−0.29, −0.09]	−0.27 [−0.37, −0.16]	0.84 [0.73, 0.90]	0.99 [0.89, 1.00]
London 2012	men's 10 m	−0.32 [−0.41, −0.24]	−0.24 [−0.34, −0.14]	0.02 [−0.35, 0.38]	−0.12 [−0.56, 0.38]
London 2012	men's 3 m	−0.25 [−0.32, −0.18]	−0.18 [−0.27, −0.08]	0.20 [−0.03, 0.41]	0.12 [−0.24, 0.46]
Rio 2016	women's 10 m	−0.00 [−0.11, 0.10]	−0.02 [−0.13, 0.10]	0.19 [−0.35, 0.63]	0.05 [−0.79, 0.83]
Rio 2016	women's 3 m	0.00 [−0.11, 0.11]	0.16 [0.02, 0.29]	0.65 [0.28, 0.85]	0.56 [0.09, 0.82]
Rio 2016	men's 10 m	−0.23 [−0.31, −0.15]	−0.27 [−0.36, −0.18]	−0.03 [−0.36, 0.31]	−0.39 [−0.66, −0.04]
Rio 2016	men's 3 m	−0.36 [−0.46, −0.25]	−0.38 [−0.46, −0.28]	0.31 [0.08, 0.51]	−0.25 [−0.55, 0.10]

Of course, as discussed earlier, any correlations may be affected by the fact that judges score lower for more difficult dives. I therefore carried out the above between- and within-rounds analyses again, this time using partial correlations to control for the current dive's difficulty. The results are presented in table 2 (second and fourth columns of values) and show that within-rounds contrast effects remain unchanged for the most part. For between-rounds scores, there is no consistent pattern and many effects show large confidence intervals. Problematically, there are far fewer between-rounds scores to analyse for each judge—only five for men's events and four for women's. In addition, because of the unchanging order of divers for each round for a given event, the between-rounds scores always involve the same two countries, e.g. Great Britain always dives last in the round in the 2016 women's 10 m platform, and USA always dives next, at the start of the following round. As such, no conclusions should be drawn with any confidence based on these between-rounds results without additional data.

## 4. Discussion

This research has investigated sequential effects in the judging of synchronized dives across four different events over two Olympic Games. In the majority of cases, I found strong evidence of contrast effects—the previous diving pair in a sequence had a negative influence on the scores of the current pair of divers if the previous divers performed well. However, athletes' scores benefited if they competed after a diving pair that performed poorly. This bias is particularly undesirable for any diving pair who has trained for many years, only to find themselves performing after the event's favourites, simply because of a random position allocation.

The current findings show opposite patterns to several previous studies. First, I found serial position effects, where divers who competed later in the competition received lower scores (cf. [7]). Here, the explanation appears to be that later dives involve a higher degree of difficulty, and this results in lower-scoring dives. However, this pattern also contradicts previous findings (cf. [23]). Although it is not clear why gymnasts receive higher execution scores for more difficult performances while divers receive lower scores, it may be a sport-specific effect. Perhaps, the process of judging, along with the determination of difficulty (judged in gymnastics by a panel during the routine), produces opposite effects in the two types of event. Further exploration across additional sports may help to shed some light on this difference.

Sequential judgements in the current data show a contrast effect rather than assimilation (cf. [9]) or no bias (cf. [7]). Previous research has focused on gymnastics competitions and this may provide an explanation for the differing pattern of results found here. When gymnasts compete, judges appear to naturally consider the similarities between athletes [9], and this may lead to the observed assimilation in scores across performances. While divers also demonstrate similarities in appearance, demographic profiles, etc. perhaps the event's focus on synchronization is the key. Judges are required to consider how well the divers perform as a pair. Although they may quantify this in terms of ‘how similar were the two divers?’ (which should lead to assimilation), it may be more likely that their approach is one of ‘how did the divers differ?’ (focusing on contrast). The latter would see judges looking for mistakes and deducting points rather than spotting similarities and adding points. At such a high level of competition, one would predict that near-perfect performances are judged more often in this way, although this has yet to be considered experimentally.

Timing may also play a role in the effects found here. Although dives only last a few seconds, this duration might be considered long enough to result in contrast effects rather than assimilation [16]. However, previous research has demonstrated that scores are assimilated in Olympic gymnastics [9], where the majority of routines/events take far longer than the time that divers spend in the air. Perhaps more in line with prediction, I find weaker sequential effects (if any) for between-rounds analyses, where the time interval between dives is longer than that found within the rounds. As with ratings of essays [11], the influence of the preceding dive may decrease with a longer between-dive interval. If this were true, scoring at events where time pressures are less significant (e.g. non-televised competitions) and the intervals between dives can be longer, may result in weaker or no sequential effects.

The conclusion that even Olympic judges, who are highly qualified and experienced [34], demonstrate significant biases while scoring elite-level performances may seem surprising. For certain types of bias, there are procedures in place to limit their influence. For example, judges who show a nationalistic bias (giving higher scores to athletes of the same nationality as themselves) will only have a limited effect on overall scores, given that many scoring systems involve the exclusion of the highest and lowest score(s) prior to calculation of the final score. For sequential effects, organisers have sensibly randomized the order of the competitors for the first round of the competition. This suggests that sports authorities may have identified the biasing influence of previous divers on judges' scores and have decided upon randomization as the best solution. Unfortunately, the dive order remains constant over subsequent rounds following the first. Therefore, if a diving pair is unlucky enough to compete after a pair that is performing well in the first round, they will continue to dive directly after that pair for all remaining rounds (with continuing negative consequences for their scores). Assuming sequential effects are difficult to remove through instruction, training, etc. perhaps the best solution might be to randomize the order of divers on every round. Of course, this brings its own disadvantages in terms of, for instance, the differing amounts of preparation time between dives.

Given these biases that seem inherent in judgements of sequences, one method of tackling the issue might be to remove the human element altogether by automating the process. Automatic diving analysis using computational techniques, once sufficiently advanced/reliable, could make the whole process of judging these types of events one of objective measurements [35,36]. This would bring competitions involving judgements of abilities into line with events where performances are already judged automatically (e.g. 100 m sprinting times or faults in tennis using the Hawk-Eye system). Although the technology is not yet suitable, this line of research represents a promising solution for the future, either as a stand-alone system or one that augments the current judging process.

To conclude, Olympic judges show contrast effects when scoring synchronized divers. This bias causes a decrease in scores for athletes that follow a high-scoring pair, and an increase in scores for those who follow a low-scoring pair. This pattern of results is the reverse of the assimilation in scores found with gymnasts, demonstrating that effects appear to be sport-specific rather than a general result of judging athletes in sequence. Such a bias represents an unfair (dis)advantage for divers that I recommend the Fédération Internationale de Natation (swimming's governing body) address in the coming years. More broadly, this type of bias may be common in some form across many sports and requires urgent consideration if the goal is to remove these influences on athletes' sporting careers.

## Supplementary Material

Judges' scores for all synchronised diving events at the 2012 and 2016 Olympic Games

## Data accessibility

The datasets supporting this article have been uploaded as part of the electronic supplementary material.
